# Empathy, burnout, life satisfaction, correlations and associated socio-demographic factors among Chinese undergraduate medical students: an exploratory cross-sectional study

**DOI:** 10.1186/s12909-019-1788-3

**Published:** 2019-09-06

**Authors:** Qinghua Wang, Lie Wang, Meng Shi, Xuelian Li, Rong Liu, Jie Liu, Min Zhu, Huazhang Wu

**Affiliations:** 1 English Department, School of Fundamental Sciences, China Medical University, No. 77 Puhe Road, Shenyang North New Area, Shenyang, Liaoning Province People’s Republic of China; 2 Department of Social Medicine, School of Public Health, China Medical University, No. 77 Puhe Road, Shenyang North New Area, Shenyang, Liaoning Province People’s Republic of China; 3 Department of Epidemiology, School of Public Health, China Medical University, No. 77 Puhe Road, Shenyang North New Area, Shenyang, Liaoning Province People’s Republic of China; 4 Department of Statistics, School of Public Health, China Medical University, No. 77 Puhe Road, Shenyang North New Area, Shenyang, Liaoning Province People’s Republic of China; 5 Department of Healthcare Management, School of Humanities and Social Sciences, China Medical University, No. 77 Puhe Road, Shenyang North New Area, Shenyang, Liaoning Province People’s Republic of China

**Keywords:** Empathy, Burnout, Life satisfaction, Medical students

## Abstract

**Background:**

Medical education is widely known to be a demanding process that may cause various mental health problems, such as burnout, which can lead to lowered levels of life satisfaction among medical students. Research shows that empathy is negatively correlated with burnout, but there are few studies on the relationship among empathy, burnout and life satisfaction in medical students. The objective of the present study is to explore the correlations of empathy and burnout with life satisfaction and the associated socio-demographic factors among Chinese undergraduate medical students.

**Methods:**

In this cross-sectional study, 1271 undergraduate medical students (age 19.42 ± 1.34 years, 36% male) from 1st to 4th grades completed questionnaires including the Interpersonal Reactivity Index Chinese version (IRI-C), the Maslach Burnout Inventory Modified Chinese version (MBI-MC), the Satisfaction With Life Scale (SWLS) and socio-demographic characteristics. Statistical analyses included Student’s t-test, one-way ANOVA, post hoc Bonferroni tests, hierarchical linear regression analysis and general linear model-univariate full factorial model.

**Results:**

Over four academic years, medical students’ empathy levels declined, but their burnout levels almost plateaued and their life satisfaction levels witnessed an initial fall before a rebound. Empathy was correlated with students’ age and grade, and burnout was associated with students’ maternal education. Significant differences in life satisfaction were detected with regard to medical students’ age, academic year, the number of children in the family, place of residence and parents’ educational levels.

**Conclusions:**

Empathy explained 0.6% of the variance in life satisfaction in contrast to 13.7% of the variance explained by burnout in life satisfaction. Although empathy did not have a main effect on life satisfaction, there was an interaction effect of empathy and burnout on life satisfaction among students of high and low empathy and burnout levels. Students with high levels of empathy and low levels of burnout were most satisfied with life. Medical institutions and related authorities need to find effective measures to enhance students’ empathy levels and reduce burnout to improve their life satisfaction.

## Background

It is widely acknowledged that medical professionals are subjected to heavy workloads, long work hours, a stressful work environment, high job pressure, and an intense physician-patient relationship, which lead to various mental health problems, such as anxiety, depression and burnout. These psychological problems exert detrimental effects on the life quality and life satisfaction of health professionals, which in turn impact the quality of care they provide to patients [1, 2].

One often cited factor related to occupational burnout among medical professionals is compassion fatigue, which is closely associated with empathy [3, 4]. Empathy refers to the ability to stand in others’ shoes, to understand as well as to share other people’s emotions and feelings and the ability to communicate this understanding [5]. Empathy is an essential element in the physician-patient relationship and affects clinical outcomes, patient compliance and satisfaction [6, 7]. Therefore, great importance is attached to empathy in undergraduate medical education, the critical phase in nurturing competent physicians, and the development of empathy in medical students is considered a goal in medical education [8].

The literature indicates that occupational burnout may have its root in the medical schools where health professionals were trained as students. In addition to stress, anxiety and depression, burnout is known to be another mental health problem prevalent in medical students. Burnout is defined as a syndrome of prolonged emotional exhaustion, depersonalization (cynicism) and a feeling of professional inefficacy [9]; possible reasons that medical students are prone to burnout problems are the intensive medical curriculum, lack of social support and inappropriate coping styles. Studies show that burnout can occur from the outset of undergraduate medical education during preclinical years [10], and the problem becomes more serious with the increase of grade levels [11].

The negative association of empathy with burnout among medical professionals is recorded in a large number of studies [1–4, 12–15]. Regarding the correlation of empathy and burnout among medical students, cross-sectional multi-institutional research conducted in Brazil concluded that personal accomplishment, one facet of the Maslach Burnout Inventory (MBI), held the strongest negative association with personal distress, which is a subscale of the Interpersonal Reactivity Index (IRI) [16]. Another study with a sample of 265 third-year medical students found that empathy measured by the Jefferson Scale of Empathy (JSE) was positively correlated with personal accomplishment while negatively associated with depersonalization, which are two subscales of the MBI [17]. Lucchetti and colleagues conducted cross-cultural empirical research comparing the differences between American and Brazilian medical students in terms of mental health, life quality, empathy and burnout. The results indicated that US medical students scored significantly higher in empathy as measured by the Empathy, Spirituality, and Wellness in Medicine survey (ESWIM) than their Brazilian counterparts whereas American medical students were more likely to suffer from exhaustion as measured by the Oldenburg Burnout Inventory (OLBI) [18].

Studies consistently showed that burnout is significantly negatively related to life satisfaction among medical students and health professionals. Life satisfaction, as a concept concerning the cognitive aspect of subjective wellbeing, is defined as an individual’s conscious evaluation of his or her life based on self-set standards [19]. The research conducted among 452 pharmacy students from five South Korean universities using structural equation modelling (SEM) found that exhaustion and cynicism, which are two facets of the Maslach Burnout Inventory—Student Survey (MBI-SS), were both significantly negatively correlated with life satisfaction [20]. A three-wave seven-year prospective study among 3255 Finnish dentists revealed that burnout predicted depressive symptoms and lowered life satisfaction [21]. Demerouti and colleagues detected a significantly negative relationship between exhaustion and disengagement, which are two facets of the Oldenburg Burnout Inventory (OLBI), and life satisfaction among 109 German nurses [22]. Although previous studies have confirmed the associations among empathy, burnout and life satisfaction, to the best of our knowledge, there has been no study exploring the possible interaction effect of empathy and burnout on life satisfaction in medical students. Additionally, whereas some previous studies have examined the associated socio-demographic factors such as age, gender and grade with empathy, burnout and life satisfaction, no research has examined the correlations of socio-demographic factors such as place of residence and parents’ educational levels with empathy, burnout and life satisfaction simultaneously in Chinese undergraduate medical students. Therefore, the aims of the present study were to (i) investigate the associated socio-demographic factors with empathy, burnout and life satisfaction and (ii) determine whether there is an interaction effect of empathy and burnout on life satisfaction in Chinese undergraduate medical students.

## Methods

### Study design and subjects

This cross-sectional study was conducted at China Medical University, which is a major medical university in Northeast China. A stratified random cluster sampling method was adopted, and questionnaires were distributed from September to November 2018 to whole classes of medical students in their 1st to 4th academic year of studies. The purpose of the survey was explained beforehand, and participation was voluntary. Written informed consent was obtained from every subject, and the study was approved by the China Medical University Committee on Human Experimentation.

### Measurement of empathy

The two most widely used measurement scales of empathy are the Jefferson Scale of Empathy (JSE) [23] and the Interpersonal Reactivity Index (IRI) [24]. Considering the large number of preclinical student participants who had little or no contact with real patients in this study, we used the IRI to measure empathy. With permission from the author of the IRI Mark H. Davis, Zhan [25], a scholar from Taiwan, translated the IRI scale bi-directionally and tested the validity as well as reliability of the IRI Chinese version and adapted the original 28-item scale into the 22-item IRI scale Chinese version (IRI-C). The IRI-C included four subscales: Perspective Taking (PT: 5 items), Personal Distress (PD: 5 items), Fantasy Scale (FS: 6 items) and Empathic Concern (EC: 6 items). Each item was scored on a 5-point Likert scale from 0 (not true of me at all) to 4 (very true of me). Rong and colleagues [26] tested the IRI-C in Chinese college students and demonstrated the sound psychometric properties of the scale. The Cronbach’s alpha coefficients for the four subscales in the present study were PT: 0.767, PD: 0.806, FS: 0.665, EC: 0.615 and the Cronbach’s alpha coefficient for the total scale was 0.760.

### Measurement of burnout

The Maslach Burnout Inventory Modified Chinese version (MBI-MC) was adapted by Lian [27], which was the most widely used burnout inventory among college students in China. Based on the MBI, the MBI-MC reduced the 22 items in the original scale to a 20-item scale that contained three subscales: Low Mood (LM: 8 items), Improper Behaviour (IB: 6 items) and Low Achievement (LA: 6 items). Example questions in the MBI-MC are “I feel exhausted after studying for a whole day” and “I can calmly handle my emotional problems”. Each item was scored on a 5-point Likert scale from 1 (not true of me at all) to 5 (very true of me), and after negatively worded questions were reverse scored, the sum score was calculated to measure medical students’ overall level of burnout. The Cronbach’s alpha coefficients for the three subscales in the present study were LM: 0.828, IB: 0.702, LA: 0.664 and the Cronbach’s alpha coefficient for the total scale was 0.872.

### Measurement of life satisfaction

Medical students’ levels of life satisfaction were measured by the Satisfaction With Life Scale (SWLS) [19], which consisted of 5 items scored on a 7-point Likert scale. Each item received a score ranging from 1 (strongly disagree) to 7 (strongly agree), and the total score was calculated to indicate the students’ overall level of life satisfaction. The Chinese version of the scale demonstrated good validity and reliability among medical students in previous studies [28, 29], and the Cronbach’s alpha coefficient for the scale in the present study was 0.882.

### Demographic characteristics

Demographic information included participants’ gender, age, grade, race, the number of children in the family, place of residence and parents’ educational levels. Students were categorized into two age groups: 17–19 and 20–24 years old. With regard to Chinese culture, race was divided into five groups: Han, Hui, Man, Wei and Other. The number of children in the family was dichotomized into only child or not. Place of residence fell into three categories: cities, towns, and villages. Parents’ education was divided into three levels: primary school and below, secondary school, college and above.

### Statistical analysis

Student’s t-test and one-way ANOVA were used to examine the differences in empathy, burnout and life satisfaction within the categorical demographic variables. Post hoc Bonferroni tests were performed to examine if the difference was significant between every two groups concerning continuous variables with regard to multi-categorical demographic variables. Hierarchical linear regression analysis was utilized to explore the association of empathy and burnout with life satisfaction. General linear model-univariate full factorial model was used to calculate main effects, the interaction effect and simple effects of two independent variables of empathy and burnout on the dependent variable of life satisfaction. All the statistical tests were performed using SPSS 22.0 with the significance level set at *p* < 0.05 (two-tailed).

## Results

### Demographic characteristics and differences in empathy, burnout and life satisfaction

The differences in the continuous variables of empathy, burnout and life satisfaction within the socio-demographic categorical variables of the subjects are presented in Table 1. Of the 1563 invited medical students, 1271 agreed to participate, signed the written informed consent forms and completed the questionnaires, with a response rate of 81.3%. The average age of the participants was 19.42 ± 1.34 years old, and the number of males was 458 (36%), while the number of females was 813 (64%). As can be seen from Table 1, the empathy level of students aged 17–19 was significantly higher than that of students aged 20–24 (52.78 vs 51.10, *p* = 0.005), and students of various grades saw a significant difference in terms of their IRI-C scores (*p* < 0.001). Burnout levels were associated with medical students’ maternal education (*p* = 0.005). Regarding life satisfaction, students aged 17–19 were more satisfied with life than those aged 20–24 (22.99 vs 22.11, *p* = 0.018), and students who were the only child in the family saw higher levels of life satisfaction (23.28 vs 21.67, *p* < 0.001). Significant differences were also observed in life satisfaction with regard to medical students’ academic year (*p* = 0.028), race (*p* = 0.044), place of residence (*p* < 0.001), paternal education (*p* < 0.001) and maternal education (*p* < 0.001).

**Table 1:** Demographic characteristics and differences in empathy, burnout and life satisfaction (*N* = 1271)

Variables	N (%)	IRI-C (M ± SD)	p	MBI-MC (M ± SD)	p	SWLS (M ± SD)	p
Gender			0.116		0.091		0.052
Male	458 (36.0)	51.44 ± 10.02		53.30 ± 12.18		22.14 ± 6.78	
Female	813 (64.0)	52.40 ± 10.70		52.13 ± 11.63		22.88 ± 6.44	
Age			0.005		0.697		0.018
17–19	725 (57.0)	52.78 ± 10.60		52.66 ± 11.55		22.99 ± 6.27	
20–24	546 (43.0)	51.10 ± 10.23		52.40 ± 12.21		22.11 ± 6.93	
Academic year			0.001		0.751		0.028
First year	415 (32.7)	53.66 ± 10.12		52.33 ± 10.92		23.33 ± 6.20	
Second year	341 (26.8)	51.73 ± 10.91		53.15 ± 12.98		22.26 ± 6.43	
Third year	264 (20.8)	51.83 ± 10.41		52.30 ± 11.25		21.91 ± 6.78	
Fourth year	251 (19.7)	50.10 ± 10.15		52.35 ± 12.31		22.67 ± 7.05	
Race			0.417		0.979		0.044
Han	1030(81.0)	52.00 ± 10.62		52.56 ± 12.05		22.50 ± 6.64	
Hui	18 (1.4)	48.06 ± 8.71		51.22 ± 9.16		24.00 ± 5.58	
Man	108 (8.5)	52.17 ± 9.66		52.28 ± 11.53		24.13 ± 6.29	
Wei	29 (2.3)	52.69 ± 7.76		52.52 ± 8.44		23.45 ± 5.58	
Other	86 (6.8)	53.26 ± 10.69		53.06 ± 11.30		21.50 ± 6.41	
The only child			0.078		0.302		< 0.001
Yes	745 (58.6)	51.62 ± 10.40		52.26 ± 11.95		23.28 ± 6.56	
No	526 (41.4)	52.67 ± 10.54		52.96 ± 11.67		21.67 ± 6.48	
Place of residence			0.214		0.209		< 0.001
Cities	609 (47.9)	52.08 ± 10.58		51.98 ± 12.45		23.53 ± 6.49	
Towns	313 (24.6)	51.28 ± 10.53		52.77 ± 11.35		22.50 ± 6.77	
Villages	349 (27.5)	52.71 ± 10.20		53.35 ± 11.13		21.12 ± 6.27	
Paternal education			0.789		0.334		< 0.001
Primary school and below	133 (10.4)	52.60 ± 10.65		53.95 ± 11.53		20.48 ± 6.65	
Secondary school	719 (56.6)	51.93 ± 10.58		52.48 ± 11.69		22.47 ± 6.36	
College and above	419 (33.0)	52.10 ± 10.24		52.22 ± 12.18		23.54 ± 6.75	
Maternal education			0.099		0.005		< 0.001
Primary school and below	187 (14.7)	53.57 ± 10.40		54.77 ± 10.83		20.46 ± 6.32	
Secondary school	706 (55.6)	51.75 ± 10.55		52.61 ± 11.71		22.55 ± 6.39	
College and above	378 (29.7)	51.87 ± 10.30		51.32 ± 12.40		23.80 ± 6.77	
Total sample	1271 (100.0)	52.06 ± 10.47		52.55 ± 11.84		22.62 ± 6.57	

Significance level: *p* < 0.05 (two-tailed); M ± SD: mean ± standard deviation; *IRI-C* Interpersonal Reactivity Index Chinese version, *MBI-MC* Maslach Burnout Inventory Modified Chinese version, *SWLS* Satisfaction With Life Scale

### Significant differences in empathy, burnout and life satisfaction within socio-demographic multicategorical variables

The significant differences in empathy, burnout and life satisfaction with regard to socio-demographic multicategorical variables were examined by ANOVA post hoc Bonferroni tests. Before the tests, the trial on equality of variance was performed to guarantee that equal variance was achieved, and the results of Bonferroni tests show that the empathy level of 1st^_^year medical students was significantly higher than that of 4^th_^year students (53.66 vs 50.10, *p* < 0.001). For burnout levels, students whose mothers held college and above qualifications were less likely to suffer from burnout problems than students whose mothers’ educational level was primary school and below (51.32 vs 54.77, *p* = 0.003). In terms of life satisfaction, 1st^_^year medical students enjoyed life more than their 3^rd_^year counterparts (23.33 vs 21.91, *p* = 0.035). Though one-way ANOVA shows that students of different ethnic groups saw different levels of life satisfaction (*p* = 0.044), no significant differences were detected by post hoc Bonferroni tests. Regarding place of residence, students from cities and towns saw higher life satisfaction levels than those from villages (M_cities_ = 23.53 vs M_villages_ = 21.12, *p* < 0.001; M_towns_ = 22.50 vs M_villages_ = 21.12, *p =* 0.020). Both maternal and paternal educational levels were correlated with medical students’ life satisfaction, with students whose parents held college and above qualifications enjoying life most (M_paternal education (college and above)_ = 23.54 vs M_paternal education (secondary school)_ = 22.47, *p* = 0.023; M_paternal education (college and above)_ = 23.54 vs M_paternal education (primary school and below)_ = 20.48, *p* < 0.001; M_maternal education (college and above)_ = 23.80 vs M_maternal education (secondary school)_ = 22.55, *p* = 0.008; M_maternal education (college and above)_ = 23.80 vs M_maternal education (primary school and below)_ = 20.46, *p* < 0.001). Students whose parents held secondary school qualifications enjoyed higher life satisfaction levels than those whose parents’ educational levels were primary school and below (M_paternal education (secondary school)_ = 22.47 vs M_paternal education (primary school and below)_ = 20.48, *p* = 0.004; M_maternal education (secondary school)_ = 22.55 vs M_maternal education (primary school and below)_ = 20.46, *p* < 0.001).

### The trend in mean scores of empathy, burnout and life satisfaction of undergraduate medical students over four academic years

**Fig. 1:**
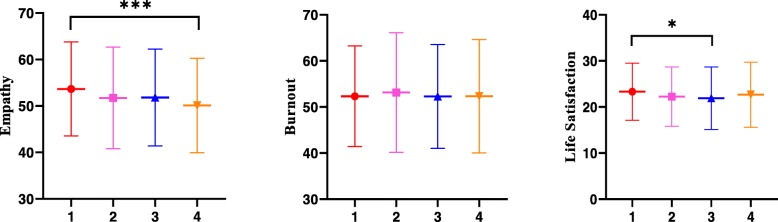
The trend in mean scores of empathy, burnout and life satisfaction of undergraduate medical students over a span of four academic years (presented as mean ± standard deviation). Total sample: *N* = 1271; 1: first year students (*n* = 415); 2: second year students (*n* = 341); 3: third year students (*n* = 264); 4: fourth year students (*n* = 251). *p < 0.05; ***p < 0.001

Figure 1 shows the trend of the 1^st^ to 4th^_^year medical students’ mean scores in empathy, burnout and life satisfaction. As revealed in the figure, there was an overall downward trend in empathy, with a significant difference detected between the 1st and 4^th^ grades (53.66 vs 50.10, *p* < 0.001). As for burnout, there was almost a leveling out since no significant differences were observed between students of various academic years. Life satisfaction saw another pattern, with the mean scores showing an initial fall to the lowest in 3^rd_^year students before rising in 4^th_^year students to a level comparable to that of 1^st_^year students. Overall, 1^st_^year medical students enjoyed life most and their mean score in life satisfaction was significantly higher than the score of 3^rd_^year students (23.33 vs 21.91, *p* < 0.05).

### Results of hierarchical linear regression analysis

Hierarchical linear regression analysis was adopted to explore the correlation of empathy and burnout with life satisfaction and the results were presented in Table 2. As can be seen from the table, demographic factors of age and gender explained 0.9% of the variance in life satisfaction. After empathy was added in step 2, the F value for the model was still significant (F = 6.287, *p* < 0.001) with empathy significantly positively predicting life satisfaction (β = 0.075, *p* < 0.01), which accounted for another 0.6% of the variance in life satisfaction. The association between burnout and life satisfaction was significantly negative (β = − 0.371, *p* < 0.001), explaining additional 13.7% of the variance in life satisfaction. It is noticeable that in all three models age was significantly negatively associated with life satisfaction (β = − 0.078, *p* < 0.01 in step 1, β = − 0.071, *p* < 0.05 in step 2 and β = − 0.074, *p* < 0.01 in step 3, respectively).

**Table 2:** Hierarchical linear regression analysis with life satisfaction as the dependent variable

Variables	Step 1 (β)	Step 2 (β)	Step 3 (β)
Step 1			
Age	−0.078^**^	−0.071^*^	−0.074^**^
Gender	0.053	0.050	0.033
Step 2			
Empathy		0.075^**^	0.053^*^
Step 3			
Burnout			−0.371^***^
F	5.833^**^	6.287^***^	56.592^***^
R^**2**^	0.009	0.015	0.152
**△**R^**2**^	0.009	0.006	0.137

Note. *N* = 1271. ^*^p < 0.05 (two-tailed); ^**^p < 0.01 (two-tailed); ^***^p < 0.001 (two-tailed)

### Main effects and the interaction effect of empathy and burnout on life satisfaction

General Linear Model-Univariate Full Factorial Model was adopted to examine the main effects and to explore whether there was an interaction effect of empathy and burnout on life satisfaction. We adopted two factors with two levels of factorial design in our study, and since there was no standard for cut-off points, we used the lower and upper quartiles to dichotomize the levels of empathy and burnout into low (< 25%) and high (> 75%). Before the test, homogeneity of variance was checked by one-way ANOVA, and the result shows that *Levene Statistic* = 0.959, *p* = 0.412, so homogeneity of variances was achieved. Table 3 demonstrates the results of between-subjects effects tests, and as revealed in the table, burnout had a main effect on medical students’ life satisfaction (*F*(1,351) = 87.008, *p* < 0.001, η_p_^2^ = 0.199). Post hoc Bonferroni tests of the main effect revealed that life satisfaction levels of students with low burnout were significantly higher than those with high burnout levels (M_life satisfaction (low burnout)_ = 25.66 vs M_life satisfaction (high burnout)_ = 19.39, *p* < 0.001) (for details, please refer to the Additional file 1). Whereas the main effect of empathy on life satisfaction did not reach significance level (*F*(1,351) = 0.362, *p* = 0.548, η_p_^2^ = 0.001), there was an interaction effect of empathy and burnout on life satisfaction (*F*(1,351) = 8.195, *p* = 0.004, η_p_^2^ = 0.023). The whole model explained 21.3% of the variance in life satisfaction (adjusted R^2^ = 0.213).

**Table 3:** Main effects and the interaction effect of empathy and burnout on life satisfaction

Source	SS	df	MS	F	p	ηp^2^
Corrected Model	3893.306^a^	3	1297.769	32.906	< 0.001	0.220
Intercept	177,449.242	1	177,449.242	4499.334	< 0.001	0.928
Empathy	14.268	1	14.268	0.362	0.548	0.001
Burnout	3431.491	1	3431.491	87.008	< 0.001	0.199
Empathy * Burnout	323.191	1	323.191	8.195	0.004	0.023
Error	13,843.088	351	39.439			
Total	199,599.000	355				
Corrected Total	17,736.394	354				

^a^R Squared = .220 (Adjusted R Squared = .213); Dependent variable: life satisfaction; *SS* type III sum of squares, *df* degree of freedom, *MS* mean square, *η*_*p*_^*2*^ partial eta squared. Effect size was measured by partial eta squared: small = 0.01 to 0.06, medium = 0.06 to 0.138, large> 0.138

### Simple effects of two levels of empathy and burnout on life satisfaction

**Fig. 2:**
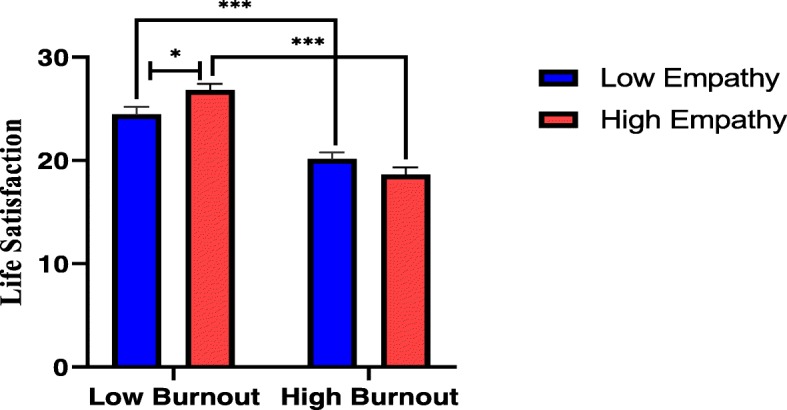
Simple effects of two levels of empathy and burnout on life satisfaction

Post hoc Bonferroni tests were performed to explore the simple effects of two levels of empathy and burnout on life satisfaction. The results of pairwise comparisons (for details, please refer to the Additional file 1) show that at the low burnout level, the mean difference in life satisfaction between students of low empathy and high empathy was significant (24.49 vs 26.82, *F*(1,351) = 5.970, *p* = 0.015, η_p_^2^ = 0.017), while at the high burnout level, the mean difference in life satisfaction between students of low empathy and high empathy was nonsignificant (20.15 vs 18.63, *F*(1,351) = 2.569, *p* = 0.110, η_p_^2^ = 0.007). When the level of empathy was fixed at low, the mean difference in life satisfaction between students of low burnout and high burnout was significant (24.49 vs 20.15, *F*(1,351) = 20.705, *p* < 0.001, η_p_^2^ = 0.056). It is noticeable that when the level of empathy was fixed at high, there was a statistically significant difference with a larger effect size in the mean value of life satisfaction between low burnout and high burnout students (26.82 vs 18.63, *F*(1,351) = 75.008, *p* < 0.001, η_p_^2^ = 0.176). Figure 2 shows the simple effects of two levels of empathy and burnout on life satisfaction by post hoc Bonferroni tests.

## Discussion

This is the first study examining the associated socio-demographic factors with empathy, burnout and life satisfaction and the possible interaction effect of empathy and burnout on life satisfaction in Chinese undergraduate medical students. As for the correlation of empathy with socio-demographic characteristics, it is found that younger and lower-grade students were more empathetic than their older higher-grade counterparts. This finding is consistent with the conclusion in a study by Li and colleagues [30], but in contradiction with the result of another study by Wen [31]. It is worth mentioning that although the sample in these two studies was Chinese medical students, the scale used for measuring empathy was the student version of the Jefferson Scale of Physician Empathy (JSPE-S). Research results concerning empathy in medical students from other cultures were also inconsistent. A study with a sample of 320 medical students across the 1^st^ to 6th academic years in Brazil detected no significant differences in empathy considering students’ grade [32], whereas research in Iran indicated that the overall level of empathy among medical students in the preclinical period was higher than that in the clinical period [33]. However, another study from India showed that medical students’ empathy scores had a tendency to decline initially and then rebound over time [34]. The contradiction in these findings may be due to the differences in measurement tools, cultures and the environment in which the empirical research was carried out. Another point worth mentioning is that no significant difference in the mean empathy score was found between males and females in the present study, which was in line with the conclusion of the study by Li [30], although a number of other studies revealed that female medical students were more empathetic than males [31–34].

A new finding in the present study was that medical students’ burnout levels were related to maternal but not paternal educational levels, and students whose mothers held college and above qualifications were less likely to suffer from burnout problems than students whose mothers’ educational level was primary school and below. In China, as in most other countries around the world, mothers still play a major role in children’s growth and education and spend relatively more time with children in comparison to fathers. Research shows that maternal education is inextricably correlated with children’s language skills, academic performance, intellectual functioning and maladaptive behaviours [35–37]. Therefore, we assume that more adaptive coping strategies were likely to be adopted by medical students whose mothers were of higher educational levels when they encountered difficulties and felt exhausted. This problem-based coping strategy, along with adjusted behaviours may help students recover from academic and emotional fatigue; thus, they experienced less burnout. The finding that no significant difference was detected in the burnout levels of students in different academic years was in line with the research conducted in 22 medical schools in Brazil [16], which concluded that burnout problems existed in all stages of medical education.

Regarding life satisfaction, students of the 17–19 age group enjoyed life more than those in the 20–24 age group. This finding is inconsistent with the conclusion in Shi [29], which did not show significant differences in the life satisfaction levels of Chinese medical students in different age groups. It is noteworthy that students in the 17–19 age group also had higher levels of empathy in the present study, and since empathy was found to be negatively correlated with stress and burnout but positively associated with social support [16, 38], it is reasonable that students with higher levels of empathy also enjoyed higher levels of life satisfaction. In the current study, 1^st_^year medical students had significantly higher life satisfaction scores than 3^rd_^year students but not 4th -year students, which can be explained from the perspective that the 3rd academic year in China is the period in which undergraduate medical students transitioned from preclinical to clinical studies, and this critical transitional period may bring maladjustment and mental health problems such as stress, anxiety and depression that lead to decreased life satisfaction. In the 4th academic year, medical students gradually adapt to the curriculum and learning environment, such as internships in university-affiliated hospitals, so the levels of life satisfaction rebound. As expected, students who were the only child in the family, who were from cities and towns and whose parents were of higher educational levels enjoyed higher levels of life satisfaction since they were more likely to have better living conditions and receive better education. It is worth mentioning that the difference in life satisfaction was marginally significant with regard to gender in this study, with females more satisfied with life than their male counterparts (22.88 vs 22.14, *p* = 0.052). This finding can be explained by the one-child policy in effect in China for several decades; under this policy, boys were more valued and parental expectations for them were higher, and compared with females, males shouldered more pressure and responsibilities, as they were considered pillars of the family, so they were more likely to suffer from mental health problems such as depression, which was demonstrated in some studies of Chinese medical students [39, 40], and this may lead to lower life satisfaction in males.

Hierarchical linear regression analysis shows that burnout explained a large proportion of the variance (13.7%) in life satisfaction, whereas empathy only accounted for 0.6% of the variance in life satisfaction. Age was found to significantly negatively predict life satisfaction and this result was in line with the finding in the present study that students of the 17–19 age group were more satisfied with life than those in the 20–24 age group. With the increase of age and grade, medical students need to deal with more academic work, especially when they started their clinical phase study. The maladjustment brought by the transitional period from preclinical to clinical learning environment and the heavy workload may result in decreased life satisfaction levels.

The results of ANOVA show that burnout had a main effect on life satisfaction, and to effectively address burnout problems, we need to find stressors that lead to burnout in medical students. First, the intensive curriculum and the rigid scoring system in medical education were found to be a source of stress. Slavin and colleagues [41] instituted curricular changes including a pass/fail grading system and reduced contact hours for preclinical students and found that there was a significant decline in medical students’ stress, anxiety and depression levels. Second, learning environments such as clinical rotation factors for clinical students and personal characteristics and life events were significant contributors to burnout. A study by Dyrbye [42] detected the associations of call frequency and clinical workload in rotation with burnout among medical students in their clinical practice environment, and the study also concluded that personal life events, gender and learning environment were all factors associated with burnout among preclinical students. Third, lack of social support and low empathy levels as well as dysfunctional coping strategies were detected in some studies as risk factors for burnout [43, 44]. It is worth mentioning that, in the research by Mazurkiewicz and colleagues, control over daily schedule and one’s self-efficacy were also found to be related to medical students’ burnout [10]. In addition to reforming the medical curriculum and scoring system, improving the learning environment, promoting social support and functional coping strategies, nurturing students’ resilience and empathy were all found to be effective in alleviating medical students’ stress and combating burnout problems.

Our study shows that although empathy did not have a main effect on life satisfaction, it interacted with burnout and exerted an interaction effect on the levels of medical students’ life satisfaction. Medical students with high levels of empathy and low levels of burnout enjoyed life most, and this finding sheds light on identifying feasible interventions to enhance students’ empathy and reduce burnout problems to improve their life satisfaction. It is noteworthy that the effect size was larger for the mean difference in life satisfaction in high empathy groups with low and high burnout levels than in low empathy groups with low and high burnout levels (η_p_^2^ = 0.176 vs η_p_^2^ = 0.056). This finding indicates that students with high empathy were probably more affected by burnout with regard to life satisfaction than students with low empathy, which lends further support to the idea that enhancing students’ empathy and reducing burnout need to be simultaneously addressed to meet the target of achieving high life satisfaction levels. Unlike among health professionals, empathy may not exert such a significant impact on burnout among undergraduate medical students, especially among preclinical students who have little or no contact with real patients. However, cultivating students’ empathy is critical and needs to be emphasized from the outset of medical education as this has a long-term effect. At present, some commonly known measures to enhance empathy include early exposure to clinical training, inter-professional education (IPE), a curriculum emphasizing communication and interpersonal skills, a self-reflection approach, creating a culture of humanism and teachers as role models in medical education. Since studies show that the empathy levels of medical students may erode as they step into higher grades, there is an urgent need to experiment with different methods to enhance empathy and implement effective methods in medical education.

Several limitations of the present study should be acknowledged. First, with the cross-sectional design, conclusions about causality cannot be drawn, and longitudinal studies should be carried out in the future. Second, as self-administered questionnaires were distributed to collect data, response bias and social desirability bias cannot be avoided. Third, the present study was conducted in one medical university in China, so generalizations of the conclusions should be made with caution and future multi-institutional research in different cultures is suggested. Fourth, the present study analysed the effect of empathy on burnout and life satisfaction of medical students, but in addition to empathy, other variables such as self-efficacy and self-esteem also influenced burnout and life satisfaction levels in medical students, which should be examined in future studies.

## Conclusions

This study explored the relationship among empathy, burnout and life satisfaction and the associated socio-demographic factors in Chinese undergraduate medical students. The results show that the levels of empathy were related to students’ age and grade, and levels of burnout were associated with students’ maternal education. As for life satisfaction, significant differences were detected with regard to medical students’ age, academic year, the number of children in the family, place of residence and parents’ educational levels. Over the four academic years, medical students’ empathy levels saw a decline, but their burnout levels almost plateaued, and their life satisfaction levels witnessed an initial fall before a rebound. There was an interaction effect of empathy and burnout on life satisfaction, and students with high empathy and low burnout were most satisfied with life. Medical institutions and related authorities need to find effective measures to enhance students’ empathy levels and reduce burnout problems to improve their life satisfaction.

## Supplementary information

**Additional file 1:** Data on main effects and simple effects of empathy and burnout on life satisfaction by ANOVA post hoc Boferroni tests. (DOCX 58 kb)

## Data Availability

The datasets used and/or analyzed in the present study are available from the corresponding author on reasonable request.

## Abbreviations

EC: Empathic Concern
ESWIM: Empathy, Spirituality, and Wellness in Medicine survey
FS: Fantasy Scale
IB: Improper Behaviour
IPE: Inter-professional education
IRI: Interpersonal Reactivity Index
IRI-C: Interpersonal Reactivity Index Chinese version
JSE: Jefferson Scale of Empathy
JSPE-S: student version of the Jefferson Scale of Physician Empathy
LA: Low Achievement
LM: Low Mood
MBI: Maslach Burnout Inventory
MBI-MC: Maslach Burnout Inventory Modified Chinese version
MBI-SS: Maslach Burnout Inventory-Student Survey
OLBI: Oldenburg Burnout Inventory
PD: Personal Distress
PT: Perspective Taking
SEM: structural equation modelling
SWLS: Satisfaction With Life Scale
