# Supplementary material for: Empathy, burnout, life satisfaction, correlations and associated socio-demographic factors among Chinese undergraduate medical students: an exploratory cross-sectional study
Source: BMC Med Educ. 2019 Sep 6;19:341. doi: 10.1186/s12909-019-1788-3 (PMC6729024; doi:10.1186/s12909-019-1788-3)

**TableS1** **Significant differences in empathy, burnout and life satisfaction within socio-demographic**

**multi-categorical variables by post hoc Bonferroni tests**

| Variables IRI-C MBI-MC SWLS |
| --- |

Academic year

First year

Second year  1^st^ vs 3^rd^ (p=0.035)

Third year 1^st^ vs 4^th^ (p<0.001)

Fourth year

Place of residence

Cities

Towns Cities vs villages (p<0.001)

Villages Towns vs villages (p=0.020)

Paternal education

Primary school and below ^a^  a vs b (p=0.004)

Secondary school ^b^  a vs c (p<0.001)

College and above ^c^  b vs c (p=0.023)

Maternal education

Primary school and below ^a^  a vs b (p<0.001)

Secondary school ^b^   a vs c (p=0.003) a vs c (p<0.001)

College and above ^c^ b vs c (p=0.008)

Significance level: p<0.05 (two-tailed); IRI-C: Interpersonal Reactivity Index Chinese version; MBI-MC: Maslach Burnout Inventory

Modified Chinese version; SWLS: Satisfaction With Life Scale.

**TablesS2 Main effects of two levels of empathy and burnout on life satisfaction by post hoc Bonferroni tests**

| **Estimates** | | | | |
| --- | --- | --- | --- | --- |
| Dependent Variable: Life Satisfaction | | | | |
| Empathy | Mean | Std. Error | 95% Confidence Interval | |
|  |  |  | Lower Bound | Upper Bound |
| Low | 22.323 | .477 | 21.384 | 23.261 |
| High | 22.726 | .473 | 21.797 | 23.656 |

| **Pairwise Comparisons** | | | | | | |
| --- | --- | --- | --- | --- | --- | --- |
| Dependent Variable: Life Satisfaction | | | | | | |
| (I) Empathy | (J) Empathy | Mean Difference (I-J) | Std. Error | Sig.^a^ | 95% Confidence Interval for Difference^a^ | |
|  |  |  |  |  | Lower Bound | Upper Bound |
| Low | High | -.404 | .672 | .548 | -1.725 | .917 |
| High | Low | .404 | .672 | .548 | -.917 | 1.725 |
| Based on estimated marginal means | | | | | | |
| a. Adjustment for multiple comparisons: Bonferroni. | | | | | | |

| **Univariate Tests** | | | | | | | | |
| --- | --- | --- | --- | --- | --- | --- | --- | --- |
| Dependent Variable: Life Satisfaction | | | | | | | | |
|  | Sum of Squares | df | Mean Square | F | Sig. | Partial Eta Squared | Noncent. Parameter | Observed Power^a^ |
| Contrast | 14.268 | 1 | 14.268 | .362 | .548 | .001 | .362 | .092 |
| Error | 13843.088 | 351 | 39.439 |  |  |  |  |  |
| The F tests the effect of Empathy. This test is based on the linearly independent pairwise comparisons among the estimated marginal means. | | | | | | | | |
| a. Computed using alpha = .05 | | | | | | | | |

| **Estimates** | | | | |
| --- | --- | --- | --- | --- |
| Dependent Variable: Life Satisfaction | | | | |
| Burnout | Mean | Std. Error | 95% Confidence Interval | |
|  |  |  | Lower Bound | Upper Bound |
| Low | 25.657 | .476 | 24.720 | 26.593 |
| High | 19.392 | .474 | 18.461 | 20.324 |

| **Pairwise Comparisons** | | | | | | |
| --- | --- | --- | --- | --- | --- | --- |
| Dependent Variable: Life Satisfaction | | | | | | |
| (I) Burnout | (J) Burnout | Mean Difference (I-J) | Std. Error | Sig.^b^ | 95% Confidence Interval for Difference^b^ | |
|  |  |  |  |  | Lower Bound | Upper Bound |
| Low | High | 6.265^*^ | .672 | .000 | 4.944 | 7.585 |
| High | Low | -6.265^*^ | .672 | .000 | -7.585 | -4.944 |
| Based on estimated marginal means | | | | | | |
| *. The mean difference is significant at the .05 level. | | | | | | |
| b. Adjustment for multiple comparisons: Bonferroni. | | | | | | |

| **Univariate Tests** | | | | | | | | |
| --- | --- | --- | --- | --- | --- | --- | --- | --- |
| Dependent Variable: Life Satisfaction | | | | | | | | |
|  | Sum of Squares | df | Mean Square | F | Sig. | Partial Eta Squared | Noncent. Parameter | Observed Power^a^ |
| Contrast | 3431.491 | 1 | 3431.491 | 87.008 | .000 | .199 | 87.008 | 1.000 |
| Error | 13843.088 | 351 | 39.439 |  |  |  |  |  |
| The F tests the effect of Burnout. This test is based on the linearly independent pairwise comparisons among the estimated marginal means. | | | | | | | | |
| a. Computed using alpha = .05 | | | | | | | | |

**TablesS3 Simple effects of two levels of empathy and burnout on life satisfaction by post hoc Bonferroni tests**

| **Estimates** | | | | | |
| --- | --- | --- | --- | --- | --- |
| Dependent Variable: Life Satisfaction | | | | | |
| Empathy | Burnout | Mean | Std. Error | 95% Confidence Interval | |
|  |  |  |  | Lower Bound | Upper Bound |
| Low | Low | 24.494 | .716 | 23.086 | 25.901 |
|  | High | 20.152 | .631 | 18.910 | 21.393 |
| High | Low | 26.820 | .628 | 25.585 | 28.055 |
|  | High | 18.633 | .707 | 17.243 | 20.023 |

| **Pairwise Comparisons** | | | | | | | |
| --- | --- | --- | --- | --- | --- | --- | --- |
| Dependent Variable: Life Satisfaction | | | | | | | |
| Burnout | (I) Empathy | (J) Empathy | Mean Difference (I-J) | Std. Error | Sig.^b^ | 95% Confidence Interval for Difference^b^ | |
|  |  |  |  |  |  | Lower Bound | Upper Bound |
| Low | Low | High | -2.326^*^ | .952 | .015 | -4.199 | -.454 |
|  | High | Low | 2.326^*^ | .952 | .015 | .454 | 4.199 |
| High | Low | High | 1.519 | .947 | .110 | -.345 | 3.382 |
|  | High | Low | -1.519 | .947 | .110 | -3.382 | .345 |
| Based on estimated marginal means | | | | | | | |
| *. The mean difference is significant at the .05 level. | | | | | | | |
| b. Adjustment for multiple comparisons: Bonferroni. | | | | | | | |

| **Univariate Tests** | | | | | | | | | |
| --- | --- | --- | --- | --- | --- | --- | --- | --- | --- |
| Dependent Variable: Life Satisfaction | | | | | | | | | |
| Burnout | | Sum of Squares | df | Mean Square | F | Sig. | Partial Eta Squared | Noncent. Parameter | Observed Power^a^ |
| Low | Contrast | 235.462 | 1 | 235.462 | 5.970 | .015 | .017 | 5.970 | .683 |
|  | Error | 13843.088 | 351 | 39.439 |  |  |  |  |  |
| High | Contrast | 101.328 | 1 | 101.328 | 2.569 | .110 | .007 | 2.569 | .359 |
|  | Error | 13843.088 | 351 | 39.439 |  |  |  |  |  |
| Each F tests the simple effects of Empathy within each level combination of the other effects shown. These tests are based on the linearly independent pairwise comparisons among the estimated marginal means. | | | | | | | | | |
| a. Computed using alpha = .05 | | | | | | | | | |

| **Estimates** | | | | | |
| --- | --- | --- | --- | --- | --- |
| Dependent Variable: Life Satisfaction | | | | | |
| Empathy | Burnout | Mean | Std. Error | 95% Confidence Interval | |
|  |  |  |  | Lower Bound | Upper Bound |
| Low | Low | 24.494 | .716 | 23.086 | 25.901 |
|  | High | 20.152 | .631 | 18.910 | 21.393 |
| High | Low | 26.820 | .628 | 25.585 | 28.055 |
|  | High | 18.633 | .707 | 17.243 | 20.023 |

| **Pairwise Comparisons** | | | | | | | |
| --- | --- | --- | --- | --- | --- | --- | --- |
| Dependent Variable: Life Satisfaction | | | | | | | |
| Empathy | (I) Burnout | (J) Burnout | Mean Difference (I-J) | Std. Error | Sig.^b^ | 95% Confidence Interval for Difference^b^ | |
|  |  |  |  |  |  | Lower Bound | Upper Bound |
| Low | Low | High | 4.342^*^ | .954 | .000 | 2.465 | 6.219 |
|  | High | Low | -4.342^*^ | .954 | .000 | -6.219 | -2.465 |
| High | Low | High | 8.187^*^ | .945 | .000 | 6.328 | 10.046 |
|  | High | Low | -8.187^*^ | .945 | .000 | -10.046 | -6.328 |
| Based on estimated marginal means | | | | | | | |
| *. The mean difference is significant at the .05 level. | | | | | | | |
| b. Adjustment for multiple comparisons: Bonferroni. | | | | | | | |

| **Univariate Tests** | | | | | | | | | |
| --- | --- | --- | --- | --- | --- | --- | --- | --- | --- |
| Dependent Variable: Life Satisfaction | | | | | | | | | |
| Empathy | | Sum of Squares | df | Mean Square | F | Sig. | Partial Eta Squared | Noncent. Parameter | Observed Power^a^ |
| Low | Contrast | 816.566 | 1 | 816.566 | 20.705 | .000 | .056 | 20.705 | .995 |
|  | Error | 13843.088 | 351 | 39.439 |  |  |  |  |  |
| High | Contrast | 2958.238 | 1 | 2958.238 | 75.008 | .000 | .176 | 75.008 | 1.000 |
|  | Error | 13843.088 | 351 | 39.439 |  |  |  |  |  |
| Each F tests the simple effects of Burnout within each level combination of the other effects shown. These tests are based on the linearly independent pairwise comparisons among the estimated marginal means. | | | | | | | | | |
| a. Computed using alpha = .05  **The following graph is made by SPSS representing the interaction effect of two levels of empathy and burnout on life satisfaction** | | | | | | | | | |


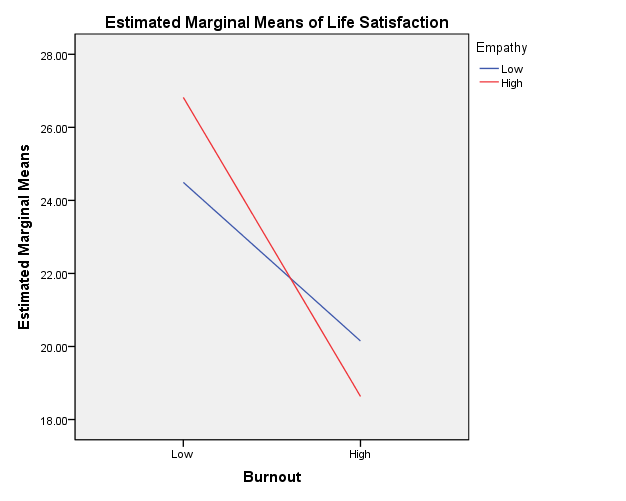

Supplement: Supplementary file 1 — Additional file 1: Data on main effects and simple effects of empathy and burnout on life satisfaction by ANOVA post hoc Boferroni tests. (DOCX 58 kb) [file 12909_2019_1788_MOESM1_ESM.docx]
